# Class 3 Obesity in a Multidisciplinary Metabolic Weight Management Program: The Effect of Preexisting Type 2 Diabetes on 6-Month Weight Loss

**DOI:** 10.1155/2020/9327910

**Published:** 2020-08-01

**Authors:** David M. Medveczky, Raymond Kodsi, Kathryn Skelsey, Kathy Grudzinskas, Flavia Bueno, Vincent Ho, Nic Kormas, Milan K. Piya

**Affiliations:** ^1^School of Medicine, Western Sydney University, NSW, Australia; ^2^South Western Sydney Metabolic Rehabilitation and Bariatric Program, Camden Hospital, NSW, Australia

## Abstract

**Introduction:**

Class 3 obesity (BMI ≥ 40 kg/m^2^) is a growing health problem worldwide associated with considerable comorbidity including Type 2 diabetes mellitus (T2DM). The multidisciplinary medical management of obesity can be difficult in T2DM due to potential weight gain from medications including sulphonylureas and insulin. However, newer weight-neutral/losing diabetes medications can aid additional weight loss. The aim of this study was to compare weight loss outcomes of patients with and without T2DM, and in patients with T2DM, to compare diabetes outcomes and change in medications at 6 months.

**Methods:**

All patients entering a multidisciplinary weight management metabolic program in a publicly funded hospital clinic in Sydney between March 2018 and March 2019, with BMI ≥ 40 kg/m^2^ and aged ≥18 years were included. Data was collected from patient clinical and electronic notes at baseline and 6 months.

**Results:**

Of the 180 patients who entered the program, 53.3% had T2DM at baseline. There was no difference in percentage weight loss in those with or without T2DM (4.2 ± 4.9% vs. 3.6 ± 4.7%, *p* = 0.35). Additionally, T2DM patients benefited from a 0.47% reduction in HbA1c (*p* < 0.01) and a reduction in the number of medications from baseline to 6 months (1.8 ± 1.0/patient vs. 1.0 ± 1.2/patient, *p* < 0.001). T2DM patients who started on weigh-neutral/losing medications in the program lost more weight than those started on weight-gaining medications (7.7 ± 5.3% vs. 2.4 ± 3.8%, *p* = 0.015).

**Conclusions:**

Patients with class 3 obesity had significant weight loss at 6 months in this program. Patients with T2DM at baseline had comparable weight loss at 6 months, a significant improvement in glycaemic control, and a reduction in diabetes medication load. Additionally, patients with T2DM who were started on weight-neutral/losing medications lost significantly more weight than those started on weight-gaining medications, and these medications should be preferentially used in class 3 obesity and comorbid T2DM.

## 1. Introduction

Obesity, defined as body mass index (BMI) ≥ 30 kg/m^2^, is a growing health problem with over a third of the global population overweight or obese [1], and this is projected to rise above 50% by 2030 [2]. Prevalence of class 3 obesity (BMI ≥ 40 kg/m^2^) has risen precipitously by an estimated 76.5% from 2000 to 2012 [3] and is strongly associated with increased mortality [4]. Obesity is associated with significant medical comorbidity including type 2 diabetes mellitus (T2DM), cardiovascular disease (CVD), obstructive sleep apnoea (OSA), gastroesophageal reflux disease (GORD), cancer, and arthritis [5, 6]. T2DM is highly prevalent in class 3 obesity with one study reporting 15.5% and 20.5% new T2DM diagnoses in patients with a BMI of 40–49.9 kg/m^2^ and ≥50 kg/m^2^, respectively [7]. Complications of T2DM make up a huge proportion of the economic burden of comorbidity, as it has large economic effects across low income as well as developed countries, and account for 1.8% of global GDP (projected to rise to up to 2.2% in 2030) [8, 9]. Furthermore, complications of T2DM have detrimental effects on health-related quality of life [10].

Class 3 obesity and T2DM are linked by several pathophysiological mechanisms including insulin resistance, inflammatory cytokines, altered lipid metabolism, and other cellular processes [11]. T2DM and obesity confer additive risk, as patients with T2DM are more likely to develop cardiovascular disease (CVD) as their BMI increases [12]. Similarly, amelioration of obesity and T2DM is closely linked, as weight loss of 5-10% in T2DM has been shown to confer a reduction of 0.6-1% in HbA1c and other metabolic benefits [13]. Furthermore, results of the DiRECT trial using lifestyle measures for T2DM showed a clear correlation between weight loss and remission in recent onset T2DM, with 86% remission in those who lost ≥15 kg at 1 year [14].

Bariatric surgery has been shown to be very effective for weight loss in class 3 obesity with a significant number also achieving T2DM remission [15]. However, bariatric surgery services are limited and are currently unable to fully cater for the scope of the obesity epidemic [16]. Furthermore, determining fitness for surgery requires a complex, multidisciplinary assessment with consideration of significant postoperative demands [17]. Thus, many patients may opt for nonsurgical weight loss programs including medical and lifestyle management, even though weight loss outcomes are not as impressive [18]. The Look AHEAD trial showed significant weight loss of 6% in its intensive lifestyle arm at 9.6-years median follow-up time [19], despite being stopped prematurely due to no evidence of cardiovascular benefit [20]. Multidisciplinary medical management including lifestyle modification has also been shown to achieve short-term weight loss and T2DM remission in severely obese populations [21, 22]. Pharmacotherapy for obesity has been shown to have modest efficacy ranging from 0.6 kg to 5.8 kg in studies with short follow-up times (12–18 months)—however, these study populations were not restricted to class 3 obesity [23].

Physical activity is also an important aspect of the management of obesity and T2DM. Patients with T2DM who performed at least moderate levels of physical activity have been shown to have reduced all-cause mortality compared to those who performed no or mild levels of physical activity [24, 25]. Physical capacity outcomes have been shown to improve in patients with class 3 obesity enrolled in our multidisciplinary program that included supervised exercise [26]. However, patients with class 3 obesity appear to face more barriers to physical exercise than patients without class 3 obesity, and patients may decline participation in supervised exercise programs [27].

The aim of pharmacotherapy in the treatment of T2DM and comorbid class 3 obesity should be extended beyond glycaemic control to include weight management [28]. Unfortunately, balancing glycaemic control and weight status can be difficult. Many conventional diabetes medications used in T2DM including sulphonylureas, pioglitazone, and insulin can cause weight gain [28, 29]. Fortunately, the newer agents of sodium-glucose transporter 2 (SGLT-2) inhibitors and glucagon-like peptide-1 (GLP-1) agonists can promote weight loss, while dipeptidyl peptidase-4 (DPP-4) inhibitors are weight neutral [28, 29]. In a recent study, Aldekhail et al. showed that class 3 obesity and T2DM patients in a weight loss program prescribed weight-neutral/losing diabetes medications lost more weight than those prescribed weight-gaining medications [30].

In Australia, 31.3% of adults have obesity [31] and 3% have class 3 obesity [32]. Access to bariatric surgery is very limited in the public hospital system with >90% of surgeries funded privately [16]. Lih et al. showed that a novel, multidisciplinary metabolic rehabilitation program (MRP) model of care can achieve greater weight loss and reduction in HbA1c in class 3 obesity and T2DM than usual care in a diabetes outpatient clinic [21]. Therefore, this exploratory observational study set out to evaluate the short-term outcomes of weight loss and glycaemic control in a similar multidisciplinary metabolic clinic that enrols patients with class 3 obesity where the primary goal is to achieve weight loss.

Specifically, we aimed to answer the following research questions:

1. Does T2DM status at baseline influence weight loss at 6 months?
2. For patients with T2DM at baseline, did glycaemic control improve and did diabetes medication use reduce at 6 months?
3. For patients with T2DM at baseline, was diabetes medication class associated with greater weight loss at 6 months?

## 2. Materials and Methods

This study was conducted in a publicly funded, hospital-based, multidisciplinary metabolic clinic in greater Sydney. All patients enrolled were over 18 years of age with a BMI ≥40 kg/m^2^ and at least one weight-related medical comorbidity, the most common being T2DM or nonalcoholic fatty liver disease (NAFLD). The components of the metabolic clinic have been previously described in detail by Atlantis et al. [26]. In brief, the program offers a multidisciplinary approach involving endocrinologists, a gastroenterologist, dietitian, a psychiatrist, psychologists, physiotherapists and a specialist nurse. Each patient attends two group education sessions prior to attending their first medical appointment with their physician. All patients are seen by a psychologist and a dietitian, and those with diabetes see a diabetes educator (specialist nurse). Then, depending on clinical need and availability, patients are reviewed every 6–12 weeks. Notably, the use of pharmacotherapy for obesity is very limited within the metabolic program, as none of the agents are subsidised by the Pharmaceutical Benefits Scheme (PBS) in Australia. In addition, all patients are offered the opportunity to attend on-site group exercise classes supervised by a physiotherapist. Patients were encouraged to attend at least 3 sessions per week and could attend more if desired. Around half of the patients in the program opt to attend the exercise classes.

All patients first seen in the program between March 2018 and March 2019 that attended at least one medical appointment were included in this study. Exercise participation was recorded if patients attended three or more classes during the 6-month follow-up period. Detailed history and blood tests were collected at baseline and 6 months for all patients as part of routine care. Patients who were included at baseline were followed up between 4 and 8 months with the aim of recording data closest to the 6-month follow-up time. Electronic and paper records were reviewed to obtain demographic details and medical history including anthropometry, medications, and blood results.

Diabetes medication prescribing was classed as weight-neutral/losing and weight-gaining as previously described in Aldekhail et al. [30]. The weight-neutral/losing class was composed of patients on metformin only or metformin combined with a SGLT-2 inhibitor, a DPP-4 inhibitor, or a GLP-1 agonist. The weight-gaining medication class was composed of patients on a sulphonylurea, pioglitazone, or any combination of medications including insulin. Patients prescribed a combination of two classes were considered to be in a mixed-weight-effect class. The term “baseline diabetes medications” signifies the medications that patients were prescribed before entering the multidisciplinary clinic. However, this study focussed on comparing the differences in outcomes at 6 months for those who were started on the weight-gaining class of diabetes medications during their time in the program versus patients who were started on the weight-neutral/losing class of medications.

The study was approved by the South West Sydney Local Health District (SWSLHD) Quality Improvement Committee (Reference: CT22_2018) as approved by the SWSLHD Health Research Ethics Committee.

### 2.1. Data Analysis

The results were reported as the percentages and mean ± standard deviations. Differences in baseline characteristics between groups were evaluated for patients completing 6-month follow-up, using independent samples *t*-test for continuous variables and Pearson's chi-square test for categorical variables. Weight loss was converted into the percentage of body weight loss. Weight loss at 6 months was evaluated with paired *t*-tests for T2DM and non-T2DM patients. The comparison of mean percent weight loss and the proportion that lost ≥5% body weight between T2DM and non-T2DM patients was performed using independent samples *t*-testing and Pearson's chi-square testing, respectively. For patients with T2DM at baseline only, paired samples *t*-tests and McNemar tests were used to assess change in continuous and categorical variables, respectively, over 6 months. Independent samples *t*-testing was conducted to assess difference in mean percentage weight loss and changes to diabetes medication class prescribing. *P* < 0.05 was considered statistically significant. All statistical analyses were performed with SPSS version 26.

## 3. Results

**Figure 1:**
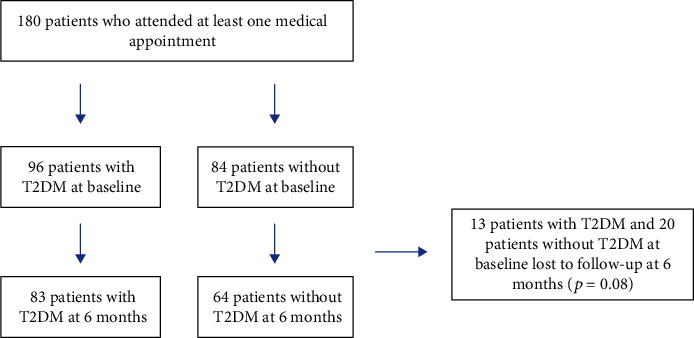
Entry of patients at baseline and loss to follow-up at 6 months, by T2DM status.

A total of 180 patients attended at least one medical appointment from March 2018 to March 2019, with 82% attending follow-up at 6 months as shown in Figure 1. Blood test results were available for 92% of patients who attended at baseline. There was no statistical difference between the proportion of patients lost to follow-up in patients with or without T2DM at baseline (13.5% vs. 23.3%, *p* = 0.08) as seen in Figure 1. Patients who were lost to follow-up were younger (mean age 44.1 ± 15 years vs. 51.0 ± 13.6 years, *p* = 0.01) and had less hypertension (45.5% vs. 69.4%, *p* < 0.01), less dyslipidaemia (45.5% vs. 65.3%, *p* = 0.03), and a history of fewer cardiac events at baseline (0 vs. 20.4%) compared to the baseline characteristics of those who were reviewed at 6 months.

Among patients who completed 6-month follow-up, at baseline, patients with T2DM weighed significantly less (137.4 ± 25.1 vs. 148.8 ± 37.8 kg, *p* = 0.038) but had no difference in BMI (50.0 ± 7.4 vs. 53.0 ± 11.0 kg/m^2^, *p* = 0.07). Patients with T2DM also had more diagnoses of hypertension (78.3% vs. 57.8%, *p* < 0.01) and dyslipidaemia (80.7% vs. 45.3%, *p* < 0.001). There were no other significant differences in the presence of comorbidities in T2DM patients compared with non-T2DM patients as shown in Table 1. The mean duration of T2DM was 9.8 ± 8.1 years. Approximately one-third of patients (31.3%) with T2DM had microvascular complications, and 19.3% had evidence of macrovascular complications at baseline. Serum cholesterol (4.2 ± 1.1 vs. 4.6 ± 1.0 mmol/L, *p* < 0.05) and LDL cholesterol (2.1 ± 1.0 vs. 2.7 ± 0.9 mmol/L, *p* < 0.001) were lower in the T2DM group, while triglycerides were higher in the non-T2DM group (2.3 ± 1.4 vs. 1.5 ± 0.7 mmol/L, *p* < 0.001). There was similar participation in the supervised exercise program between the patient groups with and without T2DM (50.6% vs. 60.9%, *p* = 0.3).

**Figure 2:**
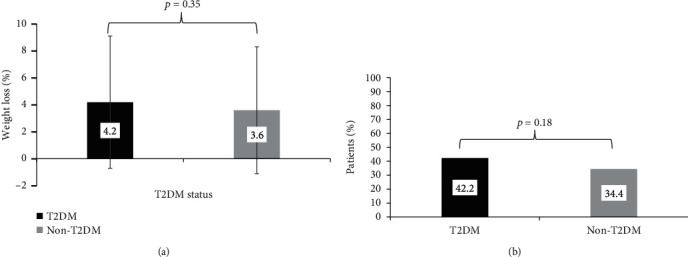
(a) Mean percent weight loss at 6 months by T2DM status at baseline (*p* = 0.35), (T2DM, *n* = 83; non-T2DM, *n* = 64). (b) Weight loss of ≥5% body weight at 6 months by T2DM status at baseline (*p* = 0.18), (T2DM, *n* = 83; non-T2DM, *n* = 64).

At 6-month follow-up, there was significant weight loss in both patients with T2DM (5.8 ± 6.8 kg, *p* < 0.001) and without T2DM (5.3 ± 7 kg, *p* < 0.001). There was no statistically significant difference in the mean percentage weight loss between patients with or without T2DM (4.2 ± 4.9% vs. 3.6 ± 4.7%, *p* = 0.35), as shown in Figure 2(a). There was also no difference between groups in the proportion of patients who lost ≥5% body weight (42.2% vs. 34.4%, *p* = 0.18) at 6 months as shown in Figure 2(b). There was no difference in the proportion of patients who lost ≥10% weight loss at 6 months in the T2DM and non-T2DM groups (10.8% vs. 6.3%, *p* = 0.33). The follow-up duration at the time of data extraction between patients with T2DM and without T2DM was similar (6.3 ± 0.9 vs. 6.2 ± 0.7 months, *p* = 0.24).

Patients with T2DM achieved a mean HbA1c reduction of 0.47% (*p* < 0.01) at 6 months, with a significantly higher proportion achieving HbA1c < 6.5% at 6 months compared to baseline (31.6% vs. 19.7%, *p* < 0.05) as shown in Table 2. At 6 months, 8.4% of patients were off all diabetes medications and had an HbA1c < 6.5%. There was an overall reduction in the number of diabetes medications from baseline to 6 months (1.8 ± 1.0/patient vs. 1.0 ± 1.2/patient, *p* < 0.001). More patients were treated with lifestyle measures alone at 6 months compared to baseline (50.6% vs. 8.4%, *p* < 0.001), and fewer patients were treated with two or more agents (61.4% vs. 30.1%, *p* < 0.001) at 6 months. Fewer patients were treated with metformin or a sulphonylurea, and patients who required insulin were treated with fewer units (total daily dose) at 6 months compared with baseline (138.4 ± 96.1 vs. 82.3 ± 47.5, *p* < 0.05).

**Figure 3:**
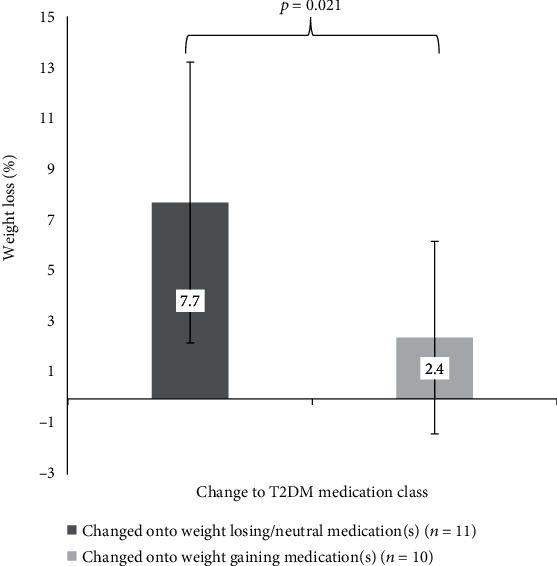
Change to diabetes medication class and weight loss at 6 months for T2DM patients.

There was a statistically significant reduction in prescribing of weight-neutral/losing diabetes medications at 6 months (43.4% vs. 24.1, *p* = 0.024), while reduction in prescribing of weight-gaining diabetes medications (34.9% vs. 21.7%, *p* = 0.071) and mixed-effect medications (13.6% vs. 3.6%, *p* = 0.057) approached significance at 6 months. Patients with T2DM who were changed onto weight-neutral/losing diabetes medications during the program lost more percent body weight than those changed onto weight-gaining medications during the program (7.7 ± 5.3% vs. 2.4 ± 3.8%, *p* = 0.02) as shown in Figure 3.

## 4. Discussion

These results from a real-world multidisciplinary weight management program demonstrate that significant weight loss, improved glycaemic control, and reduced medication load are possible in adults with class 3 obesity and T2DM at 6 months. Weight loss was achieved despite our cohort having a significantly greater burden of comorbidities compared to Australian Bariatric Surgery Registry data, including a higher prevalence of T2DM (54.6% vs. 13.6%) and higher BMI (51.3 ± 9.1 kg/m^2^ vs. 41.8 kg/m^2^) [33]. The mean BMI of our cohort (51.3 ± 9.1 kg/m^2^) is also significantly higher than pooled patient data from international systematic reviews of lifestyle interventions (37.1 kg/m^2^) and bariatric surgery (45.62 kg/m^2^) in obesity [15, 18]. The finding that older patients with more hypertension and a history of cardiovascular events were less likely to stop attending the program at 6 months may represent a greater perceived importance of the clinic by patients with greater health needs. Our cohort's higher BMI and increased comorbidity levels likely reflect higher patient selectivity by publicly funded programs due to lack of resources and patient access to multidisciplinary metabolic weight management clinics in Australia [34].

Patients with T2DM were older and had more comorbidities at baseline despite having more favourable cholesterol profiles, which was likely due to more lipid-lowering agent use seen in the T2DM patients. Despite these baseline differences, patients with T2DM achieved similar weight loss (4.2 ± 4.9 vs. 3.6 ± 4.7% body weight, *p* = 0.35) at 6 months. This finding that T2DM did not affect weight loss, in spite of a mean diabetes duration of almost 10 years, is encouraging and consistent with cohorts in an intensive medical program [22] and postbariatric surgery [35]. However, these findings conflict with earlier studies where T2DM patients fared worse after bariatric surgery [36] and in a behavioural weight-control program [37]. Anderson et al. proposed several barriers that may explain differences in weight loss for T2DM patients including genetic or metabolic predisposition to obesity, fear of hypoglycaemia, diabetes medications, weight-gaining medications used in the management of diabetes-related complications, and limitations placed on physical activity [38]. When compared to a similar metabolic rehabilitation program, patients with T2DM had comparable weight loss [21].

Specific weight goals were not recommended across the clinic, but patients agreed on realistic and achievable individualised weight goals with the clinical team. Modest weight loss was able to be achieved alongside improved glycaemic control and reduction in diabetes medication load in adults with T2DM. Furthermore, 8.4% of patients achieved an HbA1c of <6.5% while off diabetes medications at 6 months. These patients may achieve diabetes remission if they maintain this at longer-term follow-up. Our study findings are similar to the Look AHEAD trial that demonstrated that significant weight loss in T2DM (5-10%) was associated with improved glycaemic control, diabetes remission, lowered diabetes medication load, and improved fitness [19, 39, 40]. The DiRECT trial also showed that with a weight loss of 8.8 kg, patients achieved a 0.85% reduction in HbA1c, diabetes remission in 46% and a 0.8 mean reduction in diabetes medications at 12 months [14]. Despite achieving more modest weight loss (4.2 ± 4.9% for T2DM patients) in this study, it is reassuring that T2DM patients did achieve comparable reductions in HbA1c and diabetes medication load. Additionally, the HbA1c reduction of 0.47% achieved in our study is in line with results achieved by the real-world use of weight-neutral/losing medications in diabetes clinics over 12 months [41]. Furthermore, any reduction in HbA1c in overweight patients has been shown in many studies, including the UK Prospective Diabetes Study (UKPDS), to reduce long-term diabetes complications (particularly microvascular), diabetes-related death, and all-cause mortality [42]. It is therefore reassuring that the HbA1c reduction in our cohort to 7.3 ± 1.3% is likely to confer those benefits if sustained over time.

This study adds more support for prescribing weight-neutral/losing diabetes medications to patients with class 3 obesity in medical programs for weight loss. Aldekhail et al. showed that patients with T2DM prescribed a weight-neutral/losing medication lost more weight than those prescribed weight-gaining medication [30]. Our study supports this finding although we showed that changing to weight-neutral/losing medications during the program was associated with more weight loss than changing to weight-gaining medications. Reductions in medication load would be welcome for any patient with a long-term condition, and the reduction in sulphonylurea use and total daily dose of insulin is likely to lead to reduction in hypoglycaemia risk and incidence. However, there was no statistical difference in the use of weight-neutral/losing and -gaining medications at baseline compared to 6 months (24.1% vs. 21.7%). This finding likely represents a missed opportunity of pharmacotherapy-aided weight loss with newer agents in patients with T2DM. When added to metformin monotherapy, GLP-1 agonists have been shown to induce modest weight loss while DPP-4 inhibitors appear weight neutral across a large meta-analysis of RCTs [43]. SGLT-2 inhibitors have also been shown to induce weight loss compared to placebo across several meta-analyses [44], and additionally reduce cardiovascular events in patients with preexisting atherosclerotic disease [45, 46]. Guidelines updated in 2018 by the American Diabetes Association (ADA) and European Association for the Study of Diabetes (EASD) give clinicians scope to choose from several agents to combine with metformin monotherapy, and highlight weight as an important patient factor in decision-making [47]. Our findings are consistent with this message that, upon entry into a medical weight loss program, consideration should be given to the use of weight-neutral/losing diabetes medications in preference to weight-gaining medications, although cost, duration of diabetes, cardiovascular risk, and baseline HbA1c may affect choice of medication.

The finding that patients with T2DM achieved similar rates of exercise participation to patients without T2DM is encouraging, as patients with T2DM are less likely to meet national recommendations for physical activity [48], and may be impeded by autonomic/peripheral neuropathy and other disabilities [49]. Furthermore, our group has previously evaluated physical capacity outcomes in our clinic at 12 months and found improvements in several outcomes including the 6-minute walk test, which has been shown to be important for the quality of life in other disease populations [50]. Regular moderate levels of physical activity have been shown to reduce mortality in T2DM patients [24, 25], and further studies could investigate the cardiovascular benefits of exercise in class 3 obesity and comorbid T2DM.

The main strength of this study is that it was conducted in a real-world clinic that included all patients enrolled in a publicly funded hospital service with class 3 obesity and complex medical comorbidities. This could be replicated in other clinics and centres which manage patients with class 3 obesity and complex medical problems, in contrast to many clinical trials that have strict inclusion and exclusion criteria, and often exclude patients with complex medical conditions. This study was also able to access detailed clinical data and pathology, including diabetes medications prescribed. Limitations of this study include the unrecorded duration of diabetes medications taken before joining the clinic, its retrospective design, short follow-up time of 6 months, and attrition of 18.3% at 6 months, although this rate of attrition is often seen in a real-world clinic.

## 5. Conclusions

Patients with class 3 obesity and T2DM at baseline achieved similar weight loss and exercise participation to those without T2DM. In addition, patients with T2DM benefited from improved glycaemia and reduction of diabetic medication load at 6 months. These benefits support the multidisciplinary model of care for patients with class 3 obesity that is currently recommended in Australia [34]. Patients started on weight-neutral/losing diabetes medications lost more weight than those started on weight-gaining diabetes medications. Studies with longer follow-up are needed to further evaluate weight loss and glycaemic control according to diabetes medication use in class 3 obesity and comorbid T2DM.

## Figures and Tables

**Table 1:** Baseline characteristics of patients that were followed up at 6 months, by T2DM status at baseline.

Variable (mean ± SD or %)	T2DM (*n* = 83)	Without T2DM (*n* = 64)	*P* value
Age (years)	52.8 ± 13.2	48.7 ± 13.7	0.068
Females (%)	73.5	67.2	0.41
Caucasian ethnicity (%)	72.3	75.0	0.71
Weight (kg)	137.4 ± 25.1	148.8 ± 37.8	0.038
BMI (kg/m^2^)	50.0 ± 7.4	53.0 ± 11.0	0.066
Previous bariatric surgery (%)	4.2	9.5	0.27
In paid employment (%)	31.3	26.6	0.53
Exercise class participation (%)	50.6	60.9	0.21
Hypertension (%)	78.3	57.8	<0.01
Dyslipidaemia (%)	80.7	45.3	<0.001
CKD grade 3 or below (%)	15.2	13.6	0.79
Cardiovascular disease (%)	24.1	15.6	0.21
Fatty liver disease (%)	72.3	78.1	0.42
Obstructive sleep apnoea (%)	50.6	46.9	0.65
Gastroesophageal reflux disease (%)	47.0	46.0	0.91
Cholesterol (mmol/L)	4.2 ± 1.1	4.6 ± 1.0	0.038
Triglycerides (mmol/L)	2.3 ± 1.4	1.5 ± 0.7	<0.001
HDL (mmol/L)	1.1 ± 0.3	1.2 ± 0.3	0.46
LDL (mmol/L)	2.1 ± 1.0	2.7 ± 0.9	0.002
ALT (IU/L)	30.7 ± 13.9	29.1 ± 15.9	0.55

**Table 2:** Glycaemic control and diabetes medications at 6 months, for patients who had T2DM at baseline.

Variable (mean ± SD or %)	Baseline (*n* = 83)	6 months (*n* = 64)	*P* value
HbA1c (*n* = 76)			
HbA1c (%)	7.7 ± 1.7	7.3 ± 1.3	0.003
HbA1c (mmol/mol)	61.4 ± 18.5	55.7 ± 13.7	
Percentage with HbA1c < 6.5%	19.7	31.6	0.035
Number of diabetes medications per patient	1.8 ± 1.0	1.0 ± 1.2	<0.001
Type of therapy (%)			
Lifestyle only	8.4	50.6	<0.001
Monotherapy	30.1	19.3	0.18
≥2 medications	61.4	30.1	<0.001
Type of medication (%)			
Metformin	79.5	43.4	<0.001
Sulphonylurea	18.1	6.0	0.03
DPP-4 inhibitors	14.5	7.2	0.18
GLP-1 analogues	10.8	7.2	0.61
SGLT-2 inhibitors	26.5	14.5	0.087
Acarbose	1.0	0.0	N/A
Insulin	33.7	20.5	0.061
Insulin total daily dose (units)	138.4 ± 96.1	82.3 ± 47.5	0.014
Prescribing pattern (%)			
Weight-neutral/losing	43.4	24.1	0.024
Weight-gaining	34.9	21.7	0.071
Mixed-effect	13.3	3.6	0.057

## Data Availability

The data used to support the findings of this study are available from the corresponding author upon request.

## References

[1] Ng M., Fleming T., Robinson M. (2014). Global, regional, and national prevalence of overweight and obesity in children and adults during 1980–2013: a systematic analysis for the Global Burden of Disease Study 2013. *The Lancet*.

[2] Finkelstein E. A., Khavjou O. A., Thompson H. (2012). Obesity and severe obesity forecasts through 2030. *American Journal of Preventive Medicine.*.

[3] Hruby A., Hu F. B. (2015). The epidemiology of obesity: a big picture. *PharmacoEconomics*.

[4] Bhaskaran K., Dos-Santos-Silva I., Leon D. A., Douglas I. J., Smeeth L. (2018). Association of BMI with overall and cause-specific mortality: a population-based cohort study of 3·6 million adults in the UK. *The Lancet Diabetes & Endocrinology*.

[5] Pi-Sunyer X. (2002). The medical risks of obesity. *Obesity Surgery*.

[6] Ganz M. L., Wintfeld N., Li Q., Alas V., Langer J., Hammer M. (2014). The association of body mass index with the risk of type 2 diabetes: a case–control study nested in an electronic health records system in the United States. *Diabetology & Metabolic Syndrome.*.

[7] Vinciguerra F., Baratta R., Farina M. G. (2013). Very severely obese patients have a high prevalence of type 2 diabetes mellitus and cardiovascular disease. *Acta Diabetologica*.

[8] Seuring T., Archangelidi O., Suhrcke M. (2015). The economic costs of type 2 diabetes: a global systematic review. *PharmacoEconomics*.

[9] Bommer C., Sagalova V., Heesemann E. (2018). Global economic burden of diabetes in adults: projections from 2015 to 2030. *Diabetes Care*.

[10] Pham T. B., Nguyen T. T., Truong H. T. (2020). Effects of diabetic complications on health-related quality of life impairment in Vietnamese patients with type 2 diabetes. *Journal of Diabetes Research*.

[11] Eckel R. H., Kahn S. E., Ferrannini E. (2011). Obesity and type 2 diabetes: what can be unified and what needs to be individualized?. *Diabetes Care*.

[12] Fox C. S., Pencina M. J., Wilson P. W. F., Paynter N. P., Vasan R. S., D'Agostino R. B. (2008). Lifetime risk of cardiovascular disease among individuals with and without diabetes stratified by obesity status in the Framingham heart study. *Diabetes Care*.

[13] Bramante C. T., Lee C. J., Gudzune K. A. (2017). Treatment of obesity in patients with diabetes. *Diabetes Spectrum*.

[14] Lean M. E. J., Leslie W. S., Barnes A. C. (2018). Primary care-led weight management for remission of type 2 diabetes (DiRECT): an open-label, cluster-randomised trial. *The Lancet*.

[15] Chang S.-H., Stoll C. R. T., Song J., Varela J. E., Eagon C. J., Colditz G. A. (2014). The effectiveness and risks of bariatric surgery. *JAMA Surgery*.

[16] Edye M., Talbot M. L. (2014). Inequalities of access to bariatric surgery in Australia. *The Medical Journal of Australia*.

[17] Neff K., Olbers T., Le Roux C. (2013). Bariatric surgery: the challenges with candidate selection, individualizing treatment and clinical outcomes. *BMC Medicine*.

[18] Hassan Y., Head V., Jacob D., Bachmann M. O., Diu S., Ford J. (2016). Lifestyle interventions for weight loss in adults with severe obesity: a systematic review. *Clinical Obesity*.

[19] Look AHEAD Research Group (2013). Cardiovascular effects of intensive lifestyle intervention in type 2 diabetes. *The New England Journal of Medicine*.

[20] Pi-Sunyer X. (2014). The Look AHEAD trial: a review and discussion of its outcomes. *Current Nutrition Reports*.

[21] Lih A., Pereira L., Bishay R. H. (2015). A novel multidisciplinary intervention for long-term weight loss and glycaemic control in obese patients with diabetes. *Journal of Diabetes Research*.

[22] McCombie L., Brosnahan N., Ross H., Bell-Higgs A., Govan L., Lean M. E. J. (2019). Filling the intervention gap: service evaluation of an intensive nonsurgical weight management programme for severe and complex obesity. *Journal of Human Nutrition and Dietetics*.

[23] Leblanc E. S., Patnode C. D., Webber E. M., Redmond N., Rushkin M., O’Connor E. A. (2018). Behavioral and pharmacotherapy weight loss interventions to prevent obesity-related morbidity and mortality in adults. *Journal of the American Medical Association*.

[24] Blomster J. I., Chow C. K., Zoungas S. (2013). The influence of physical activity on vascular complications and mortality in patients with type 2 diabetes mellitus. *Diabetes, Obesity & Metabolism*.

[25] Sadarangani K. P., Hamer M., Mindell J. S., Coombs N. A., Stamatakis E. (2014). Physical activity and risk of all-cause and cardiovascular disease mortality in diabetic adults from Great Britain: pooled analysis of 10 population-based cohorts. *Diabetes Care*.

[26] Atlantis E., Langford K., Piya M. (2019). Physical capacity outcomes in patients with severe obesity after 12 months of physician-led multidisciplinary team care: a case series from a public hospital clinical obesity service. *Clinical Obesity*.

[27] Wiklund M., Olsén M. F., Willén C. (2011). Physical activity as viewed by adults with severe obesity, awaiting gastric bypass surgery. *Physiotherapy Research International*.

[28] Scheen A. J., Van Gaal L. F. (2014). Combating the dual burden: therapeutic targeting of common pathways in obesity and type 2 diabetes. *The Lancet Diabetes & Endocrinology*.

[29] Van Gaal L., Scheen A. (2015). Weight management in Type 2 diabetes: current and emerging approaches to treatment. *Diabetes Care*.

[30] Aldekhail N. M., Morrison D. S., Khojah H. (2020). The association between diabetes medication and weight change in a non‐surgical weight management intervention: an intervention cohort study. *Diabetic Medicine*.

[31] ABS (2018). *National Health Survey: First Results, 2017-2018*.

[32] Australian Institude of Health and Welfare (2017). *A picture of overweight and obesity in Australia 2017*.

[33] Bariatric Surgery Registry (2019). *Bariatric Surgery Registry 2018/2019 Report*.

[34] Atlantis E., Kormas N., Samaras K. (2018). Clinical obesity services in public hospitals in Australia: a position statement based on expert consensus. *Clinical Obesity*.

[35] Yip K., Heinberg L., Giegerich V., Schauer P. R., Kashyap S. R. (2012). Equivalent weight loss with marked metabolic benefit observed in a matched cohort with and without type 2 diabetes 12 months following gastric bypass surgery. *Obesity Surgery*.

[36] Schauer P. R., Burguera B., Ikramuddin S. (2003). Effect of laparoscopic Roux-en Y gastric bypass on type 2 diabetes mellitus. *Annals of Surgery*.

[37] Wing R. R., Marcus M. D., Epstein L. H., Salata R. (1987). Type II diabetic subjects lose less weight than their overweight nondiabetic spouses. *Diabetes Care*.

[38] Anderson J. W., Kendall C. W. C., Jenkins D. J. A. (2003). Importance of weight management in Type 2 diabetes: review with meta-analysis of clinical studies. *Journal of the American College of Nutrition*.

[39] (2010). The Look AHEAD Research Group. *Archives of Internal Medicine*.

[40] Look AHEAD Research Group (2014). Eight-year weight losses with an intensive lifestyle intervention: the Look AHEAD study. *Obesity*.

[41] Carls G. S., Tuttle E., Tan R.-D. (2017). Understanding the gap between efficacy in randomized controlled trials and effectiveness in real-world use of GLP-1 RA and DPP-4 therapies in patients with type 2 diabetes. *Diabetes Care*.

[42] UK Prospective Diabetes Study (UKPDS) Group (1998). Effect of intensive blood-glucose control with metformin on complications in overweight patients with type 2 diabetes (UKPDS 34). *The Lancet*.

[43] Phung O. J. (2010). Effect of noninsulin antidiabetic drugs added to metformin therapy on glycemic control, weight gain, and hypoglycemia in type 2 diabetes. *Journal of the American Medical Association*.

[44] Lee P. C., Ganguly S., Goh S. Y. (2018). Weight loss associated with sodium-glucose cotransporter-2 inhibition: a review of evidence and underlying mechanisms. *Obesity Reviews*.

[45] Zinman B., Wanner C., Lachin J. M. (2015). Empagliflozin, cardiovascular outcomes, and mortality in type 2 diabetes. *The New England Journal of Medicine.*.

[46] Zelniker T. A., Wiviott S. D., Raz I. (2019). SGLT2 inhibitors for primary and secondary prevention of cardiovascular and renal outcomes in type 2 diabetes: a systematic review and meta-analysis of cardiovascular outcome trials. *The Lancet*.

[47] Davies M. J., D’Alessio D. A., Fradkin J. (2018). Management of hyperglycemia in type 2 diabetes, 2018. A consensus report by the American Diabetes Association (ADA) and the European Association for the Study of Diabetes (EASD). *Diabetes Care*.

[48] Zhao G., Ford E. S., Li C., Mokdad A. H. (2008). Compliance with physical activity recommendations in US adults with diabetes. *Diabetic Medicine*.

[49] Marwick T. H., Hordern M. D., Miller T. (2009). Exercise training for type 2 diabetes mellitus. *Circulation*.

[50] Bohannon R. W., Crouch R. (2016). Minimal clinically important difference for change in 6-minute walk test distance of adults with pathology: a systematic review. *Journal of Evaluation in Clinical Practice*.

